# An Odontoma Found in the Wake of Maxillary Sinusitis Onset

**DOI:** 10.1155/2013/834715

**Published:** 2013-12-07

**Authors:** Megumi Sotobori, Kohei Marukawa, Masatoshi Higuchi, Ryuichi Nakazawa, Akinori Moroi, Yuri Ishihara, Ran Iguchi, Akihiko Kosaka, Hiroumi Ikawa, Koichiro Ueki

**Affiliations:** Division of Medicine, Department of Oral and Maxillofacial Surgery, Interdisciplinary Graduate School of Medicine and Engineering, University of Yamanashi, 1110 Shimokato Chuo, Chuo-shi, Yamanashi 409-3898, Japan

## Abstract

Sinusitis of dental origin is a relatively frequent entity, and odontomas are considered to be the most common odontogenic tumors of the oral cavity. Eruption and infection of odontomas are extremely rare. Here, we report an interesting case where odontoma was found in the wake of the maxillary sinusitis onset.

## 1. Introduction

Although sinusitis of dental origin is a relatively frequent entity [1], maxillary dental infections, apical leakage, migration of a tooth or root during extraction, or the presence of ectopic tooth or cyst in the sinus can also cause sinusitis [2–4]. Reports relating to odontoma are few. A case of a large compound odontoma in the right maxillary sinus causing acute maxillary sinusitis is reported [5]. Griffith and Imperato [5] say that seven large antral odontomas were identified previously; however, none had typical findings of acute sinusitis.

On the other hand, the occurrence of complex odontomas is not considered to be rare in the jaws [6–11]. Most of them are asymptomatic and are discovered during routine radiographic investigations [6, 8, 10, 12]. Moreover, they are usually associated with disturbances of neighboring teeth eruption [12, 13]. Little has been reported on odontoma related to normal tooth germs instead of supernumerary tooth germs. So, we report a patient who had odontoma associated with the second permanent molar found in the wake of the onset of maxillary sinusitis.

## 2. Case Report

**Figure 1:**
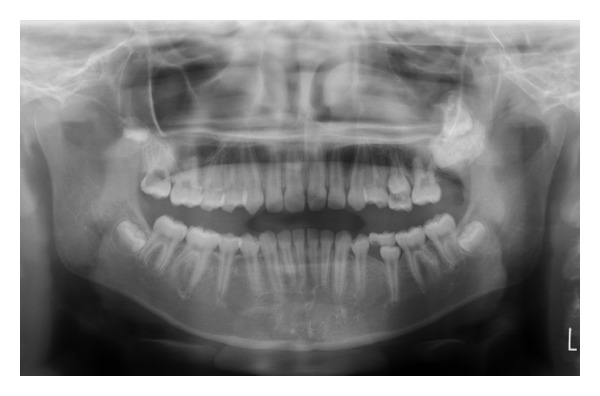
Initial panoramic radiograph.

**Figure 2:**
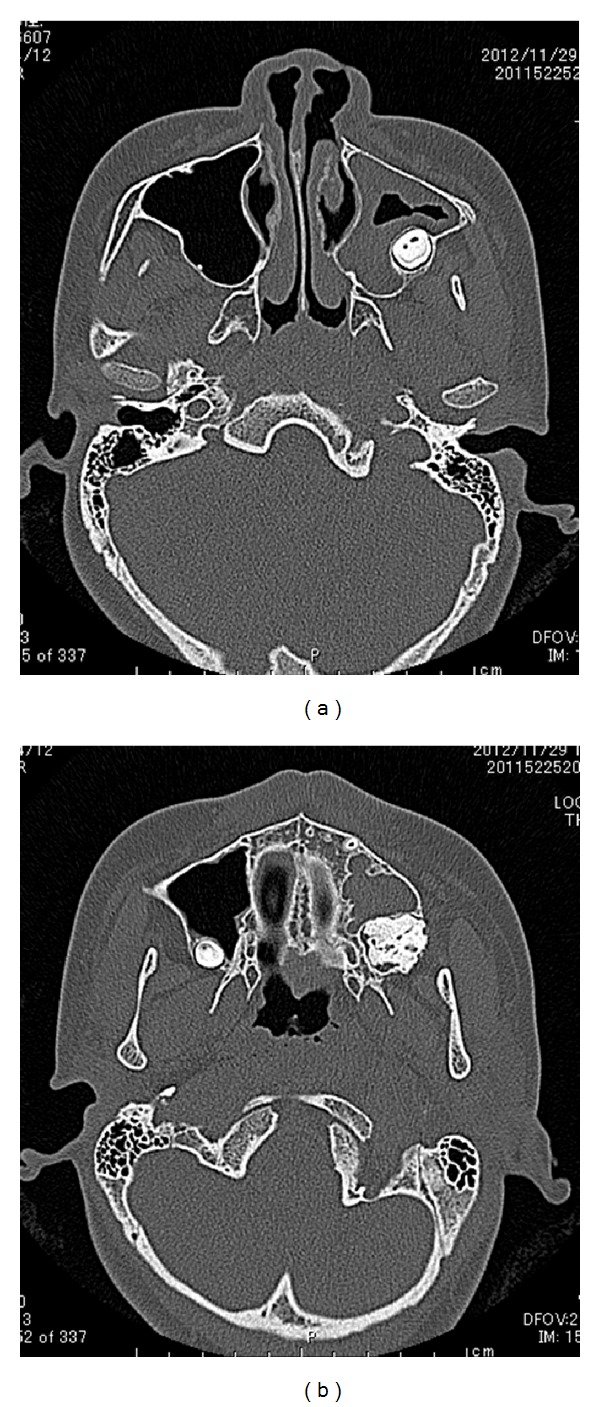
Axial CT view of the lesion.

**Figure 3:**
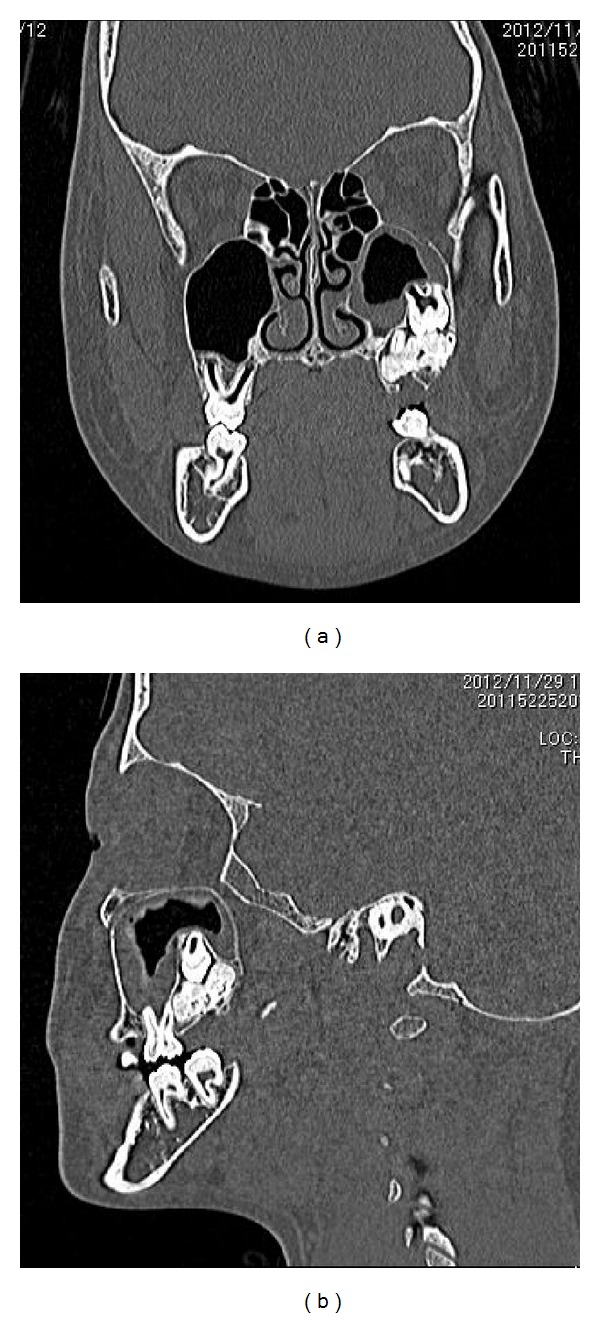
CT view of the lesion. (a) Coronal view. (b) Sagittal view.

**Figure 4:**
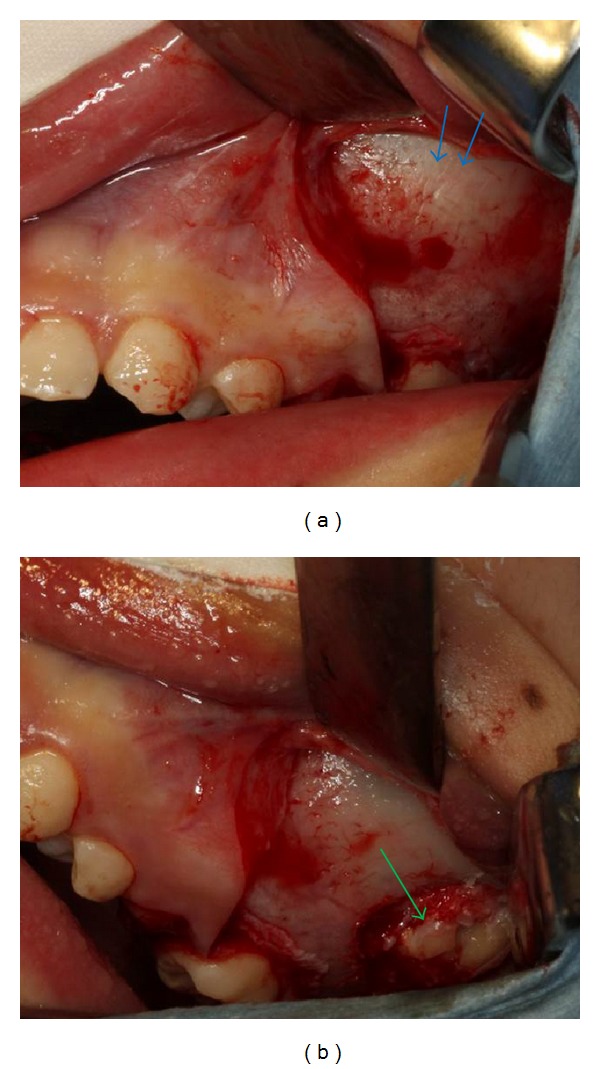
Operative findings. (a) The blue arrows show bulging and thinning of the buccal cortical bone. (b) The green arrows show a solid dental-like mass that has been impacted.

**Figure 5:**
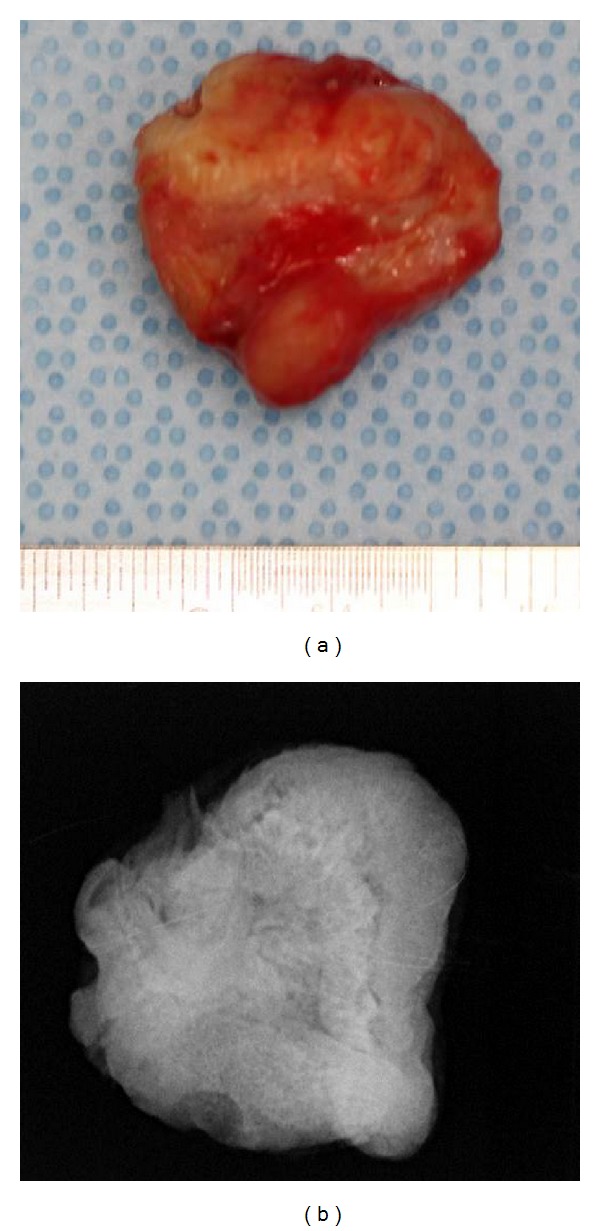
View of the excised lesion. (a) Macroscopic findings. (b) X-ray finding.

**Figure 6:**
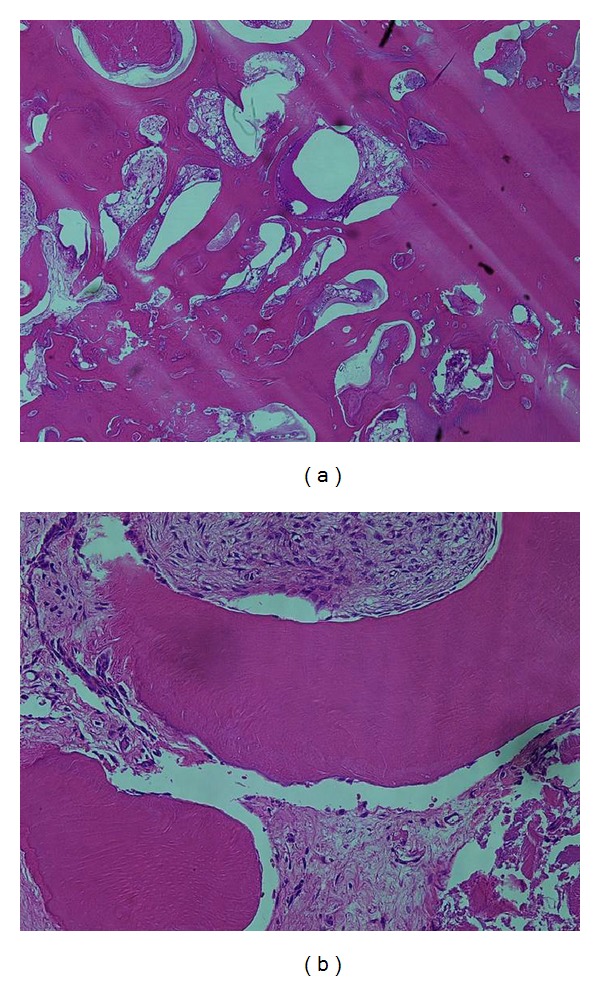
Pathological examination (hematoxylin-eosin staining). Hard tissues like dentin or enamel are mixed randomly. (a) Original magnification ×4; (b) ×20.

**Figure 7:**
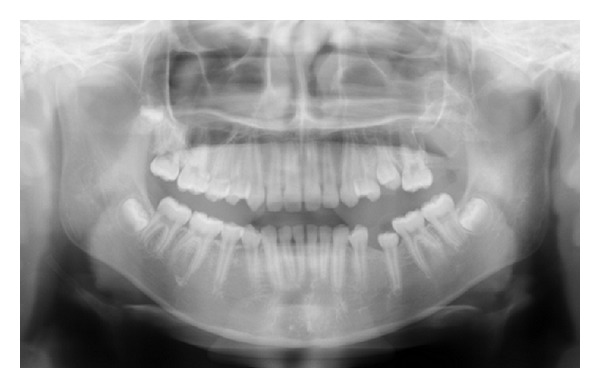
Postoperative panoramic radiograph.

**Figure 8:**
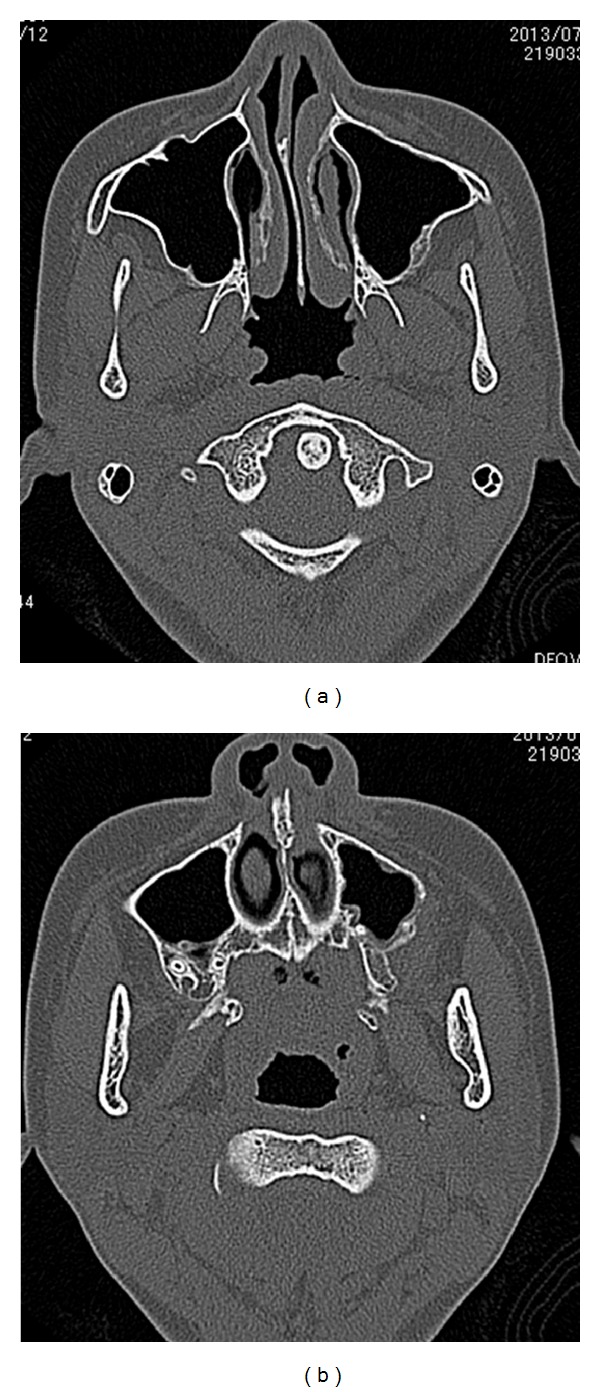
Axial CT view of the lesion.

**Figure 9:**
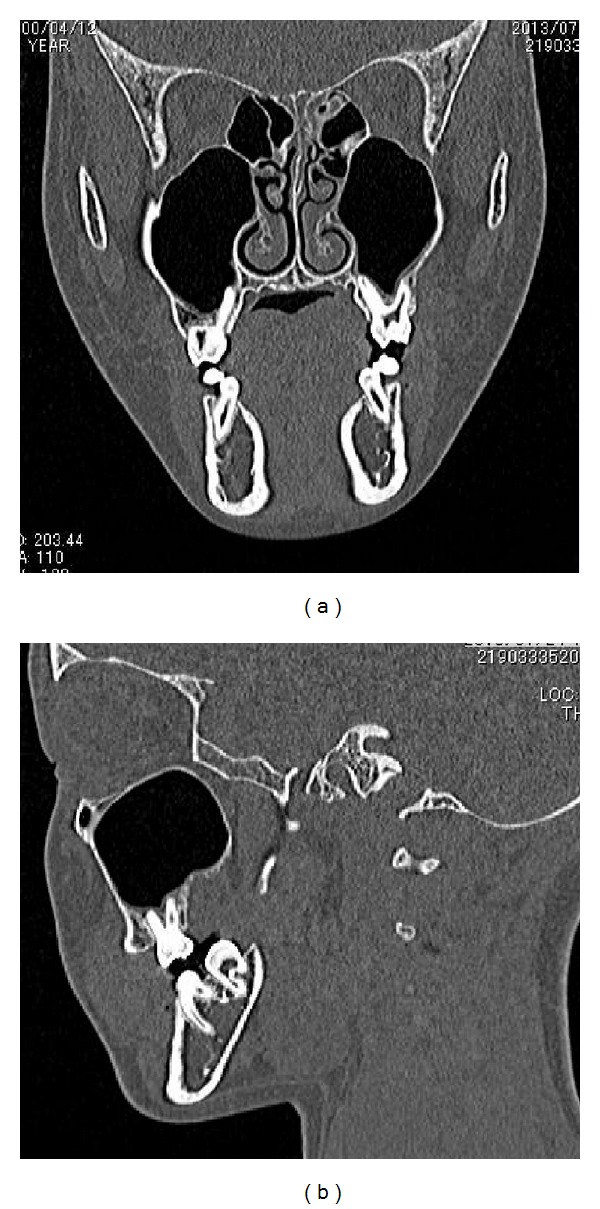
CT view of the lesion. (a) Coronal view. (b) Sagittal view.

A 12-year-and-7-month-old female pediatric patient presented with suspicious left odontogenic maxillary sinusitis and no noteworthy personal history. She complained of persistent nasal congestion and tenderness in the left molars. As a disease history, she had pain and swelling of the left cervical lymph node for one month. Symptoms were improved by intravenous infusion of antibiotics by a pediatrician who pointed out the left maxillary sinusitis by CT imaging. On suspecting odontogenic maxillary sinusitis, the patient was referred to our hospital. There was no sign of eruption of the second upper left molar; however, there was tenderness in a considerable part of the gum. Normal X-ray of the skull showed an opaque image of teeth surrounding the impaction of the second molars in the upper left maxillary sinus area (Figure 1). Computed tomography revealed an opaque image of the maxillary sinus and thickening of the maxillary sinus mucosa (Figures 2 and 3). We performed resection of the lesion under general anesthesia. When peeling the mucoperiosteal flap, slight bulging and thinning of the buccal cortical bone were noted (Figure 4(a)). Removal of the thinning bone revealed solid masses similar to dental hardening (Figure 4(b)). The lesion was removed easily as there was no adhesion to the surrounding bone and soft tissues. Moreover, there was no root exposure of the upper left first molar. The surgical specimens were 24 × 21 × 16 mm large tooth-like solid and second molar (Figures 5(a) and 5(b)). Pathologic examination revealed compound odontoma (Figures 6(a) and 6(b)). The postoperative course was good; all permanent teeth except the upper left second molar have erupted normally and with no complications or recurrence (Figure 7). Thickening of the maxillary sinus mucosa and an opaque image were not seen in the CT photo taken at 6 months after surgery (Figures 8 and 9).

## 3. Discussion

The presence of an odontoma in the sinus is exceptional [1]. Further, large odontomas involving the maxillary sinus are quite uncommon [5]. Griffith and Imperato [5] stated that there is no previous report of a large antral odontoma with the typical findings of acute sinusitis. His case of large compound odontoma in the right maxillary had severe pain and pressure. The patient was treated with antibiotics and resulted in complete remission of symptoms for several months, but the symptoms later recurred and the patient failed to respond to the symptomatic therapy. Sanders et al. [14] stated that there are only five reports of odontomas in the antrum producing symptoms of maxillary sinusitis. His case of odontoma of the right maxilla had right sinus congestion and pain. In our case, the patient complained of typical findings of persistent nasal congestion and tenderness or swelling in the left molars. Image findings also suggested maxillary sinusitis.

Odontomas are the most common odontogenic tumors [6–8, 10–13, 15] and are benign, slow growing, and nonaggressive [12, 13]. Most of them are asymptomatic and discovered during routine radiographic investigations [6, 8, 10, 12, 13, 16]. This case was found with odontoma in the wake of maxillary sinusitis symptoms such as pain and swelling. On the other hand, patients who develop odontoma secondary to long-term chronic maxillary sinusitis have also been reported [1]. Also, most of the odontomas related with supernumerary tooth germ and little odontomas are related to normal tooth germ, as follows. A 15-year-old female having maxillary anterior region's odontoma had normal eruption of the permanent teeth and completion of the root [12]. A 4-year-old female having an odontoma in the maxillary anterior region had normal eruption of the primary teeth and developed permanent tooth buds [15]. A 22-year-old male having an odontoma that extended into 2/3rd of the maxillary sinus had first and second molars that were impacted and lying above the odontoma [7]. Both the case of the 10-year-old boy with odontoma in the right maxillary anterior region [6] and that of the 14-year-old male with odontoma between the roots of 13 and 14 [17] are not associated with the normal tooth germs.

Eruption and infection of odontoma are uncommon [7, 10–12, 16], and odontomas are usually associated with tooth eruption disturbances [6, 12, 16]. Tozoglu et al. reported a case of complex odontoma that erupted into the oral cavity as a rare case [11]. Agrawal et al. stated that an unusual case of infected complex odontoma with eruption of odontoma in the oral cavity and perforation of the cheeks had a tooth that was impacted and that was the first report of such a case [16]. Though several cases of infected odontomas have been reported as rare [16, 18], infected odontoma with no eruption has not been reported yet. This case of infected odontoma in the maxillary sinus with no eruption is extremely rare.
